# A single-dose of intranasal vaccination with a live-attenuated SARS-CoV-2 vaccine candidate promotes protective mucosal and systemic immunity

**DOI:** 10.1038/s41541-023-00753-4

**Published:** 2023-10-20

**Authors:** Awadalkareem Adam, Birte Kalveram, John Yun-Chung Chen, Jason Yeung, Leslie Rodriguez, Ankita Singh, Pei-Yong Shi, Xuping Xie, Tian Wang

**Affiliations:** 1 Department of Microbiology and Immunology, University of Texas Medical Branch, Galveston, TX USA; 2 Sealy Institute for Vaccine Sciences, University of Texas Medical Branch, Galveston, TX USA; 3 Department of Biochemistry and Molecular Biology, University of Texas Medical Branch, Galveston, TX USA; 4 Institute for Human Infections and Immunity, University of Texas Medical Branch, Galveston, TX USA; 5 Sealy Institute for Drug Discovery, University of Texas Medical Branch, Galveston, TX USA; 6 Sealy Center for Structural Biology & Molecular Biophysics, University of Texas Medical Branch, Galveston, TX USA; 7 Department of Pathology, University of Texas Medical Branch, Galveston, TX USA

**Keywords:** Live attenuated vaccines, Virology

## Abstract

An attenuated SARS-CoV-2 virus with modified viral transcriptional regulatory sequences and deletion of open-reading frames 3, 6, 7 and 8 (∆3678) was previously reported to protect hamsters from SARS-CoV-2 infection and transmission. Here we report that a single-dose intranasal vaccination of ∆3678 protects K18-hACE2 mice from wild-type or variant SARS-CoV-2 challenge. Compared with wild-type virus infection, the ∆3678 vaccination induces equivalent or higher levels of lung and systemic T cell, B cell, IgA, and IgG responses. The results suggest ∆3678 as an attractive mucosal vaccine candidate to boost pulmonary immunity against SARS-CoV-2.

Severe acute respiratory syndrome coronavirus 2 (SARS-CoV-2), the cause of the coronavirus disease 2019 (COVID-19) pandemic, has resulted in over 6.9 million deaths globally over the past three years. In response to the pandemic, there has been rapid development of SARS-CoV-2 vaccines. Currently, four vaccines have been granted emergency use authorization by the FDA. Although the vaccines are highly effective against severe disease, their efficiency has been challenged by the increasing rates of variants of concern (VOCs) that are characterized by increased viral transmissibility and immune evasion^1^. Continuous work is needed to optimize existing vaccine platforms and to develop more effective novel vaccines.

Parenteral injection route is currently used for the delivery of approved COVID-19 vaccines. Such administration route triggers limited local respiratory tract immune responses, particularly against VOCs^2^. Compared to the parenteral route, delivery of antigens to the sites of infection induces mucosal immune responses in the respiratory tract, including IgA and resident memory B and T cells, which provide additional layers of protection^3^. Thus, intranasal immunization, which leads to the induction of antigen-specific immunity in both mucosal and systemic immune compartments^4^, would be more effective in controlling viral infection and disease^5,6^. Compared to hamsters, mice are relatively low in cost, easier to work with, and are most amenable to immunological manipulation. Moreover, SARS-CoV-2 infection of K18-hACE2 transgenic mice induces weight loss, interstitial pneumonitis, and lethality^7^. Thus, here, we test this hypothesis by mucosal immunization of K18-hACE2 mice with a highly attenuated SARS-CoV-2 containing modified viral transcriptional regulatory sequences and deletion of open-reading frames 3, 6, 7 and 8 (∆3678). This vaccine candidate was previously shown to protect hamsters from SARS-CoV-2 infection and transmission after a single-dose vaccination^8^. Our current study shows that a single-dose intranasal vaccination of K18-hACE2 mice with ∆3678 induces potent mucosal and systemic T cell and humoral immune responses at similar or higher levels than WT virus infection, leading to full protection of all immunized mice from WT and VOC SARS-CoV-2 challenge.

K18-hACE2 mice were immunized intranasally (i.n.) with 2 × 10^3^ PFU of ∆3678 virus based on a prior study^8^. Mice vaccinated with the wild-type (WT) SARS-CoV-2 USA-WA1/2020 or PBS (mock) were used as controls (Fig. 1a). All animals vaccinated with the ∆3678 virus or PBS survived 28 days post-vaccination (DPV) and displayed neither weight loss nor clinical signs (Fig. 1b); whereas 7.6% of the animals infected with the same dose of WT virus succumbed to disease between 7 and 12 DPV (*data not shown*). About one-third of the mice inoculated with the WT virus showed weight loss after day 7 (Fig. 1b). At 28 DPV, blood, bronchoalveolar lavage (BAL) fluid, lung, and spleen samples were collected to determine the virus-induced immune responses. In the lung, both WT and ∆3678 virus-inoculated mice displayed a Th1-prone immune response. Compared to the WT virus group, CD4^+^ T cells from the ∆3678-vaccinated mice had similar or higher levels of IFNγ^+^ production, as indicated by the percentage and total cell number increase; whereas CD8^+^IFNγ^+^ T cells displayed an increase in both percentage and total cell number (Fig. 1c, d). Both WT and ∆3678 inoculations triggered high RBD-specific IgA^+^ B cell responses, though the latter group had 25% lower response (Fig. 1e, f). Furthermore, comparable levels of SARS-CoV-2- specific IgA or IgG antibodies were detected in the BAL fluid of the WT and ∆3678 groups (Fig. 1g, h). In the periphery of WT- and ∆3678-vaccinated mice, Th1-prone immune responses were also noted in the spleen. CD4^+^IFNγ^+^ T cells in the ∆3678-vaccinated mice had similar cell numbers as the WT virus group with a decrease on the percentage, whereas CD8^+^ T cells of the ∆3678 group produced IFN-γ levels similar to the WT virus by total cell number and percentage (Fig. 1i, and Supplementary Fig. 1A). Additionally, both WT and ∆3678 viruses induced strong SARS-CoV-2- specific IgG and Th1- prone IgG2c in the sera; there was modestly lower level of IgG titers in the∆3678 group but no differences in IgG2c titers were observed between the two groups (Fig. 1j, k). Furthermore, in mice with prior exposure to SARS-CoV-2 infection, vaccination with ∆3678 induced similar or higher Th1-prone immune responses in the lung and periphery as presented by the total number of increases on CD4^+^IFNγ^+^ T cells and CD8^+^IFNγ^+^ T cells compared to WT virus -vaccinated mice. Both WT and ∆3678 viruses induced strong and comparable levels of SARS-CoV-2- specific IgG and Th1- prone IgG2c in the sera of these mice. We also noted that ∆3678-vaccination in mice with prior SARS-CoV-2 infection had stronger CD8^+^IFNγ^+^ T cell response in the lung, stronger CD4^+^IFNγ^+^ T cells and CD8^+^IFNγ^+^ T cells in the spleen, and stronger SARS-CoV-2- specific IgG2c responses in the sera compared to those without prior infection (Supplementary Fig. 1b–d). Overall, these results suggest that although ∆3678 was highly attenuated in K18-hACE2 mice, it induces potent mucosal and systemic T cell and humoral immune responses at equivalent or higher levels than WT virus in mice with or without prior SARS-CoV-2 infection.

**Fig. 1:**
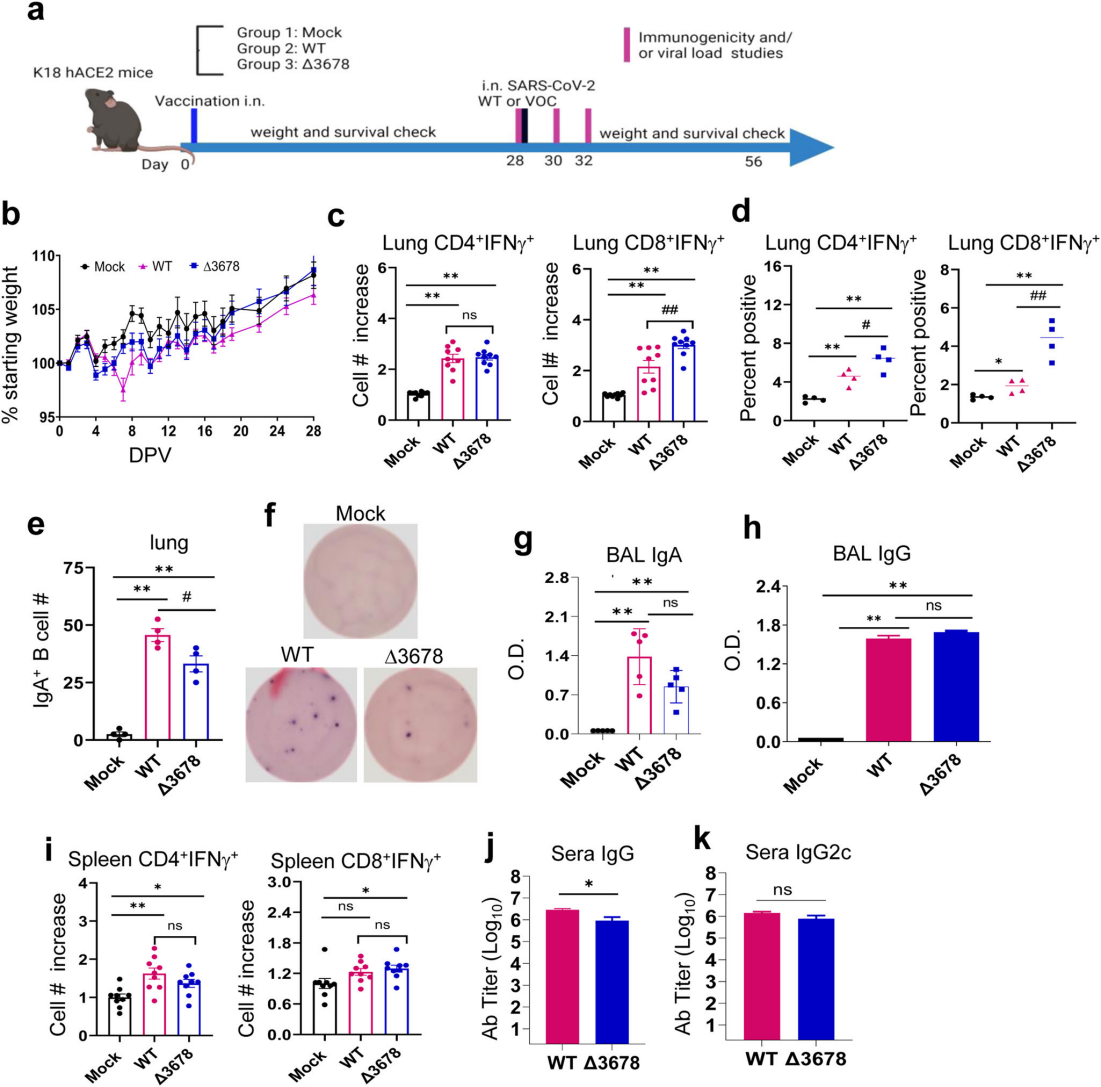
∆3678 SARS-CoV-2 induced strong mucosal and systemic immune responses in K18-hACE2 mice one month post intranasal vaccination. **a** Study design and vaccination timeline. **b** Weight loss is indicated by percentage using the weight on the day of vaccination as 100%. Lung leukocytes (**c**, **d**) or splenocytes (**i**) were cultured ex vivo with S peptide pools for 6 h, and stained for IFN-γ, CD3, and CD4 or CD8. Fold increase of IFN-γ^+^ CD4^+^ and CD8^+^ T cells expansion compared to the mock vaccinated group is shown (**c** and **i**). **d** Percent positive of IFN-γ^+^ CD4^+^ and CD8^+^ T cells among lung T cells is shown. **e**, **f** Lung leukocytes were stimulated in vitro for 7 days with immunostimulatory agents (R848 plus rIL-2) and seeded onto ELISPOT plates coated with SARS-CoV-2 RBD. Frequencies of SARS-CoV-2 RBD specific IgA-secreting lung B cells per 10^6^ input cells in MBC cultures. IgA (**g**) and IgG (**h**) titers in BAL presented as O.D. values by ELISA. Sera IgG (**j**) and IgG2C (**k**) endpoint titers by ELISA. **P < 0.01, or *P < 0.05 compared to mock group. ^##^*P* < 0.01, or ^#^*P* < 0.05 compared to WT group. Unpaired, 2-tailed Student’s *t* test was used to determine the differences. Data are presented as means ± standard error of the mean (s.e.m).

The protective efficacy of the ∆3678 mutant was determined by challenging the vaccinated mice with 10^4^ PFU of the WT USA-WA1/2020 virus on 28 DPV. Animals that had previously been inoculated with the WT or ∆3678 virus survived the challenge, while 4 out of 9 of mock-vaccinated animals succumbed to disease between days 7 and 8 post-challenge (Fig. 2a). Mice vaccinated with either virus displayed no weight loss after the challenge, while the naïve animals lost up to 20% of body weight by day 7 post-challenge (Fig. 2b). Mice that had previously been inoculated with WT or ∆3678 virus had no detectable virus in their lungs or tracheae on days 2 and 4 and significantly diminished viral loads in nasal washes at day 2 post-challenge (Fig. 2c–e). To determine protective efficacy against more recently circulating variants, we also performed a challenge with Omicron BA.5 virus—a VOC with low susceptibility to antibody neutralization when tested against previous variant infected convalescent sera or vaccinated sera^9^. Strikingly, none of the mice that had been inoculated with the ∆3678 mutant had detectable virus in the lung or trachea on day 2 post-challenge while the naïve animals had viral loads of over 10^6^ PFU/g lung tissue (Fig. 2f). Thus, vaccination with the ∆3678 mutant has equivalent protective efficacy as WT virus against subsequent WT virus or VOC challenge in mice.

**Fig. 2:**
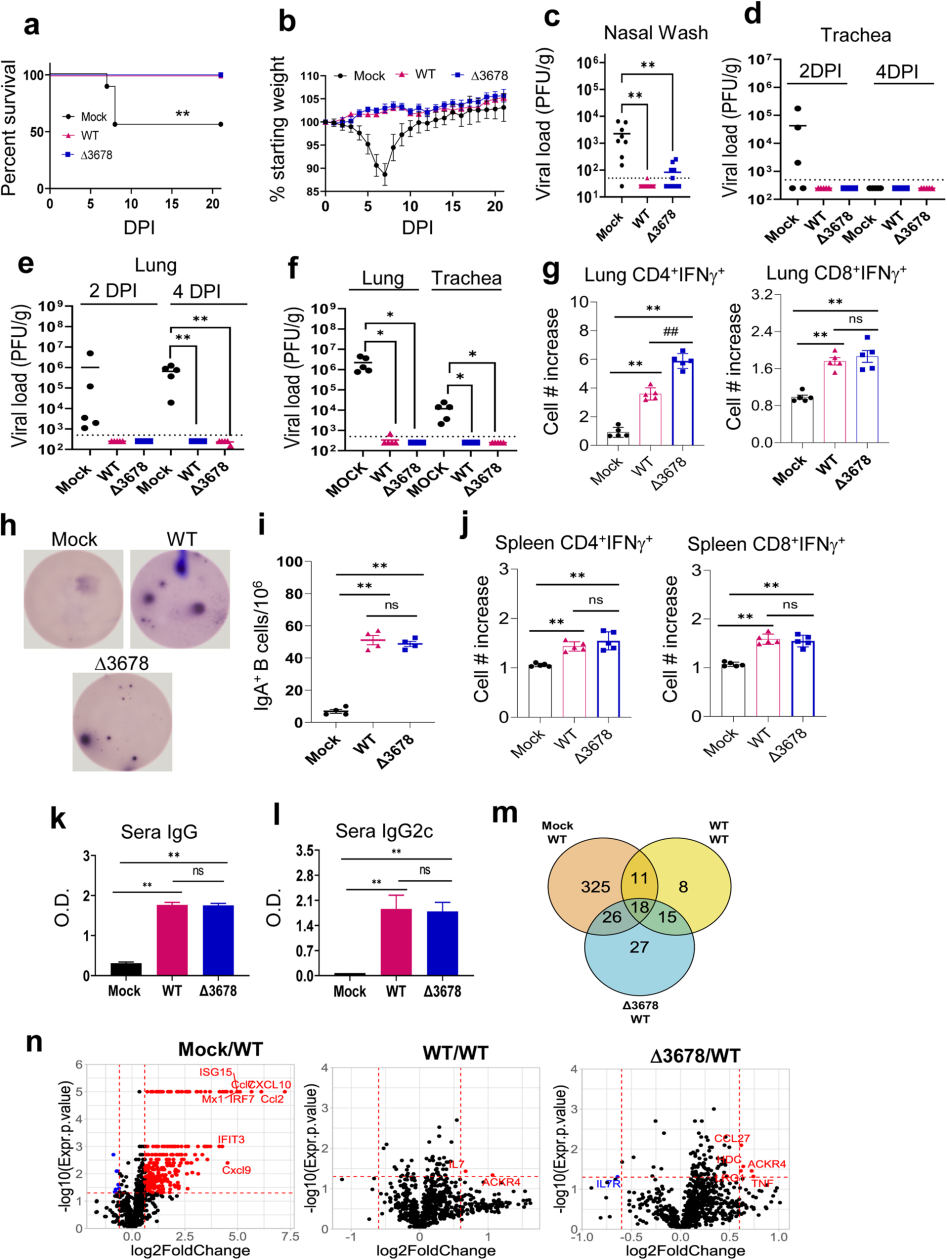
Intranasal vaccination of ∆3678 SARS-CoV-2 protects mice from WT or VOC virus challenge via induction of lung resident and systemic T cell and humoral immune responses. K18-hACE2 mice were intranasally immunized with mock (PBS), WT USA-WA1/2020 SARS-CoV-2, or ∆3678 virus. At 28 DPV, mice were intranasally challenged with 10^4^ PFU of WT USA-WA1/2020 virus. Mice were monitored daily for survival (**a**) and weight loss (**b**). Weight loss is indicated by percentage using the weight on the day of infection as 100%. **c**–**e** SARS-CoV-2 viral loads in nasal washes, trachea, and lungs were measured by plaque assays at indicated days post-infection (DPI). **f** Viral loads in trachea and lung at day 2 post-challenge with Omicron BA.5 SARS-CoV-2. Lung leukocytes (**g**) or splenocytes (**j**) were cultured ex vivo with spike peptide pools for 5 h, and stained for IFN-γ, CD3, and CD4 or CD8. Fold increase of IFN-γ^+^ CD4^+^ and CD8^+^ T cells expansion compared to the mock-vaccinated group is shown. **h**, **i** Lung leukocytes were stimulated in vitro for 7 days with immunostimulatory agents (R848 plus rIL-2) and seeded onto ELISPOT plates coated with SARS-CoV-2 RBD. Frequencies of SARS-CoV-2 RBD-specific IgA-secreting lung B cells per 10^6^ input cells in MBC cultures are shown. Sera IgG (**k**) and IgG2C (**l**) titers presented as O.D. values by ELISA. Data are presented as means ± standard error of the mean (s.e.m). **m**, **n** NanoString data for mouse lungs (harvested on day 4 post-challenge) from designated vaccination groups. All comparisons are between a challenged group and unvaccinated/unchallenged (mock/mock) controls. **m** Venn diagram of differentially expressed genes (unadjusted *p* value ≥ 0.05 from un-paired Student’s *t* test of normalized nCounter gene reads) between the three vaccination conditions. **n** Volcano plots. The horizontal dotted line corresponds to an unadjusted *p* value cutoff of 0.05 and vertical lines correspond to −0.6 and 0.6 log2 (fold change). Log-rank test (**a**) and Unpaired, 2-tailed Student’s *t* test was used to determine the differences (**c**–**n**). **P < 0.01, or *P < 0.05 compared to mock group. ^##^*P* < 0.01, or ^#^*P* < 0.05 compared to WT group.

To further assess the ∆3678-induced protective immunity, blood, lung, and spleen samples were collected on day 4 post-challenge with the WT virus. In the lung, CD4^+^ IFN-γ^+^ T cells of the ∆3678 group had about 50% increases in total cell number and percentage compared to the WT virus-vaccinated mice; while the number and percentage of CD8^+^ IFN-γ^+^ T cells were similar between the two groups. Compared to the mock group, the number of CD4^+^IFN-γ^+^ and CD8^+^ IFN-γ^+^ T cells was increased by 550 and 89% respectively (Fig. 2g and Supplementary Fig. 1E). Furthermore, ∆3678-vaccinated mice produced a similar number of IgA^+^ -expressing B cells as the WT virus-vaccinated group in response to virus challenge (Fig. 2h, i). In the periphery, splenic CD4^+^ and CD8^+^ T cells of both the WT- and ∆3678-vaccinated mice induced more IFN-γ production than the mock group at day 4 post-challenge, while equal IFN-γ levels were detected between the WT- and ∆3678-vaccinated mice (Fig. 2j and Supplementary Fig. 1F). Lastly, strong but similar titers of SARS-CoV-2- specific IgG and IgG2c antibodies were detected in the sera of both the WT- and ∆3678-vaccinated groups (Fig. 2k, l).

We next investigated the underlying immune mechanisms of ∆3678-induced host protection. By using the probe-based nCounter Analysis system, we compared the mRNA expression of 785 host genes from lung homogenates of mock-, WT-, and ∆3678-vaccinated mice at day 4 post-challenge with the WT virus. The three groups of lung homogenates were labeled mock/WT, WT/WT, and ∆3678/WT, respectively. All samples were compared with lung samples collected from the unvaccinated and unchallenged group (mock/mock). Twenty canonical pathways (Supplementary Fig. 2) were identified from comparison analysis by the Ingenuity Pathway Analysis (IPA). Among them, pathogen-induced cytokine storm signaling pathways, pyroptosis signaling pathway, necroptosis signaling pathway, and type I diabetes signaling, which are associated with the severity of SARS-CoV-2 infection^10–12^, were induced in the mock/WT group but diminished in the WT/WT and ∆3678/WT groups, suggesting that both WT and ∆3678 viruses protect host from WT virus -induced lung inflammation and pathology. Furthermore, 15 overlapping differentially expressed genes (DEGs) were observed in both WT/WT and ∆3678/WT groups (Fig. 2m, n, and Table 1). The DEGs include (i) genes associated with lung tissue-resident memory T cells (Trm) development and maintenance (e.g., *Il7* and *Tgfb3)*^13^, (ii) genes associated with CD8 Trm-initiated activities in the lung (e.g., *Gzmk*, *Fcrgrt*)^14^, and (iii) genes associated with T cell recruitment into the lung (e.g., *Ackr4*)^15^. These results further support that mucosal vaccination with the ∆3678 mutant protects mice from the SARS-CoV-2 challenge by promoting lung resident memory T cell responses.

**Table 1:** Overlapping differentially expressed genes with fold change and *p* values for each group.

	WT/WT		Δ3678/WT	
Gene	Fold Change	*P* Value	Fold Change	*P* Value
*Ackr4*	2.093	0.046	1.652	0.035
*Anpep*	1.198	0.024	1.300	0.017
*Atf4*	−1.399	0.008	−1.220	0.047
*Ccl6*	1.267	0.033	1.341	0.017
*Ctsv*	1.131	0.050	1.177	0.022
*Fcgrt*	1.314	0.022	1.333	0.024
*Gzmk*	1.332	0.022	1.384	0.049
*Il7*	1.593	0.037	1.363	0.019
*Neo1*	1.137	0.045	1.248	0.009
*Neu1*	1.137	0.017	1.115	0.043
*Os9*	1.108	0.020	1.138	0.004
*Ptpn4*	1.142	0.016	1.200	0.005
*Tgfb3*	1.072	0.018	1.180	0.042
*Timp2*	1.084	0.034	1.162	0.002
*Ywhaq*	1.102	0.007	1.107	0.002

NanoString data for mouse whole lung day 4 from designated vaccination groups. *P* values are determined by non-paired Student’s *t* tests for each gene. All comparisons are between a challenged group and unvaccinated/unchallenged (mock/mock) controls.

In summary, our results consistently indicate that ∆3678 SARS-CoV-2 is highly attenuated in K18-hACE2 mice. A single-dose of intranasal vaccination with this vaccine candidate induces potent SARS-CoV-2-specific mucosal and systemic cell-mediated and humoral immune responses in mice with or without prior SARS-CoV-2 infection and the vaccination protects host from WT and VOC virus challenge. We also demonstrated that mucosal delivery of the live-attenuated vaccine candidate promotes the development and functional activation of pulmonary Trms. Although the translatability of rodent-based intranasal vaccine research to humans remains to be determined^16^, results from this animal model provide important insights into the mucosal immunity induced by the candidate live attenuated vaccine and the correlated protective efficacy against COVID-19 infection. Overall, the ∆3678 virus may serve as an attractive candidate for future human or veterinary vaccination via the mucosal route either by itself or by a prime-pull strategy, leading to enhanced and/or durable mucosal immunity in the respiratory tracts^17,18^. Further studies on the optimization of the safety and immunogenicity of the virus and testing the protective efficacy in non-human primate models will warrant the transition to the next stage of vaccine development.

## Methods

### Viruses and cells

African green monkey kidney epithelial Vero-E6 cells (laboratory-passaged derivatives from ATCC CRL-1586) were grown in Dulbecco’s modified Eagle’smedium (DMEM; Gibco/Thermo Fisher) with 10% fetal bovine serum (FBS, HyClone Laboratories) and 1% anti-biotic/streptomycin (P/S, Gibco). Vero-E6-TMPRSS2 cells were purchased from SEKISUI XenoTech, LLC, and maintained in 10% FBS (HyClone Laboratories), and 1% P/S and 1 mg/ml G418 (Gibco). All cells were maintained at 37 °C with 5% CO_2_. The infectious clones derived USA-WA1/2020 SARS-CoV-2, ∆3678, and the BA.5 variant were generated as previously described^8,19,20^.

### Animal studies

Female heterozygous K18-hACE2 C57BL/6J mice (strain: B6.Cg-Tg(K18-ACE2)2Prlmn/J) were obtained from The Jackson Laboratory and were infected intranasally (i.n.) with 2 × 10^3^ PFU of either WT WA1 or ∆3678 infectious clone-derived viruses or were mock-infected with DPBS. Mice were between 8 and 10 weeks old at the time of initial infection and were age-matched within experimental cohorts. Intranasal challenge with 1 × 10^4^ PFU of WT WA1 or BA.5 virus was performed on day 28 after the initial infection. For each infection, animals were anesthetized with isoflurane (Piramal) and monitored regularly until fully recovered from anesthesia. Mice were monitored daily for weight changes and illness. Mice with more than 20% weight loss were euthanized by inhalation of CO_2_. At day 28 post vaccination and day 4 post virus challenge, mice were euthanized by inhalation of CO_2,_ BAL fluid, nasal washes, whole lungs, and whole spleens were collected from one subset of mice for immunological analysis. From a different subset of mice, the trachea and cranial right lobe were collected in DPBS for enumeration of viral loads via plaque assay, and the middle and caudal lobes were collected in TRIzol (Invitrogen) for RNA extraction.

### Ethic statement

All animal handling was approved by the Institutional Animal Care and Use Committee (IACUC, protocol # 2103023) of the University of Texas Medical Branch and performed under animal biosafety level 3 (ABSL3) conditions at the Galveston National Laboratory in accordance with guidelines set by the IACUC.

### Plaque assay

Collected lung lobes were homogenized in 1 ml of PBS at 6000 rpm for 60 s using a Roche MagNA Lyser instrument before titration. The lung homogenates were clarified by centrifugation at 15,000 × g for 3 min. Viral titers in the supernatants were enumeratedusing a standard plaque assay. Briefly, approximately 1.2 × 10^6^ Vero-E6-TMPRSS2 cells were seeded to each well of 6-well plates and cultured at 37 °C, 5% CO2 for 16 h. The virus was serially diluted in DMEM with 2% FBS and 200 μl diluted viruses were transferred onto the monolayers. The viruses were incubated with the cells at 37 °C with 5% CO_2_ for 1 h. After the incubation, an overlay medium was added to the infected cells per well. The overlay medium contained MEM with 2% FBS, 1% penicillin/streptomycin, and 1% Sea-plaque agarose (Lonza, Walkersville, MD). After a 2.5-day incubation, the plates were stained with neutral red (Sigma-Aldrich) and plaques were counted on a lightbox.

### Antibody ELISA

ELISA plates were coated with 100 ng/well recombinant SARS-CoV-2 RBD protein (RayBiotech) overnight at 4 °C. The plates were washed twice with PBS, containing 0.05% Tween-20 (PBS-T) and then blocked with 8% FBS for 1.5 h. Sera or BAL samples were diluted 1:100 or undiluted in blocking buffer and were added for 1 h at 37 °C. Plates were washed 5 times with PBS-T. Goat anti-mouse IgG (1:2000, 1013-05, Southern Biotech, USA), or goat anti-mouse IgG2C (1:2000, 1078-04, Southern Biotech, USA) conjugated with horseradish peroxidase (HRP) or alkaline phosphatase was added at a 1:2000 dilutions for 1 h at 37 °C followed by adding TMB (3, 3, 5, 5′- tetramethylbenzidine) peroxidase substrate (Thermo Scientific) or *p*-nitrophenyl phosphate (Sigma-Aldrich), and the intensity was read at an absorbance of 450 or 405 nm. To determine IgA titer, HRP -conjugated goat anti-mouse IgA (1:2000, 1040-05, Southern Biotech) was added as the secondary antibody at a 1:2000 dilution for 1 h at 37 °C, followed by adding TMB peroxidase substrate (Thermo Scientific) for about 15 min. The reactions were stopped by 1 M sulfuric acid, and the intensity was read at an absorbance of 450 nm. Binding endpoint titers were determined using a cutoff value which is negative control + 3x SD.

### B cell ELISPOT

ELISPOT assays were performed as previously described^21^. Briefly, Millipore ELISPOT plates (Millipore Ltd, Darmstadt, Germany) were coated with 100 µl rSARS-CoV-2 spike protein (R&D Systems). To detect total IgA^+^-expressing B cells, the wells were coated with 100 µl of anti-mouse IgA capture Ab (15 µg/ml, 3865-3, Mabtech In). Cells were added in duplicates to assess total IgA ASCs or SARS-CoV-2 specific B cells. The plates were incubated overnight at 37˚C, followed by incubation with biotin-conjugated anti-mouse IgA (0.5 µg/ml, 3865-6, Mabtech In) for 2 h at room temperature, then 100 µL/well streptavidin-ALP (1:1000) was added for 1 h. Plates were developed with BCIP/NBT-Plus substrate until distinct spots emerge, washed with tap water, and scanned using an ImmunoSpot 6.0 analyzer and analyzed by ImmunoSpot software (Cellular Technology Ltd).

### Intracellular cytokine staining (ICS)

Splenocytes or lung leukocytes were incubated with SARS-CoV-2 spike peptide pools (1 μg/ml, Miltenyi Biotec) for 6 h in the presence of GolgiPlug (BD Bioscience). Cells were stained with antibodies for CD3 (12-0031-81), CD4 (17-0041-82), or CD8 (11-0081-82) purchased from Thermo Fisher Scientific, fixed in 2% paraformaldehyde, and permeabilized with 0.5% saponin before adding anti-IFN-γ (12-7311-82, Thermo Fisher Scientific). Samples were processed with a C6 Flow Cytometer instrument. Dead cells were excluded based on forward and side light scatter (Supplementary Fig. 3). Data were analyzed with a CFlow Plus Flow Cytometer (BD Biosciences).

### Analysis of nCounter analysis system (NanoString) data

Lung samples were homogenized in Trizol (Thermo Fisher Scientific). Total RNA was purified using the Direct-zol-96 MagBead RNA kit (Zymo) with a KingFisher Apex System (Thermo Fisher Scientific). RNA samples were normalized to 20 ng/μL and followed by analysis using the nCounter Pro Analysis System and the nSolver Analysis Software. Plots were made using R version 4.1.2. An un-adjusted *p*-value cutoff of 0.05 for non-paired Student’s *t* test of normalized nCounter reads was used to determine DEGs for the three conditions. This was due to overall sample sizes and subtetly of changes in the WT/WT vs mock/mock and ∆3678/WT vs mock/mock comparisons. An additional log2 fold change cutoff of + or – 0.6 was used to limit labeling of up-and down-regulated genes in volcano plots although the Mock/WT condition had too many genes to label. The ‘ggvenn’ package version 0.1.9 was used to create Fig. 2m. IPA (version 84978992) core analysis was performed on data (gene name, un-adjusted *p*-value, and fold change) from each condition, evaluating based on expression fold changes. The option “User Data Set” was used as the reference set. Data on canonical pathways for Supplementary Fig. 2 were derived from a comparison analysis. -log(*p*-values) and z-scores were used to generate bubble plots, using ascending *p*-value or descending -log(p-value) for ordering.

### Statistical analysis

Values for viral load, cytokine production, antibody titers, B and T cell response experiments were compared using Prism software (GraphPad) statistical analysis and were presented as means ± SEM. *P* values of these experiments were calculated with a non-paired Student’s *t* test.

### Reporting summary

Further information on research design is available in the Nature Research Reporting Summary linked to this article.

### Supplementary information

SUPPLEMENTAL MATERIAL

REPORTING SUMMARY

## Data Availability

All data generated or analyzed during this study are included in this published article (and its supplementary information files).
