# Supplementary material for: A single-dose of intranasal vaccination with a live-attenuated SARS-CoV-2 vaccine candidate promotes protective mucosal and systemic immunity
Source: NPJ Vaccines. 2023 Oct 20;8:160. doi: 10.1038/s41541-023-00753-4 (PMC10589337; doi:10.1038/s41541-023-00753-4)
Supplement: Supplementary file 1 — SUPPLEMENTAL MATERIAL [file 41541_2023_753_MOESM1_ESM.pdf]

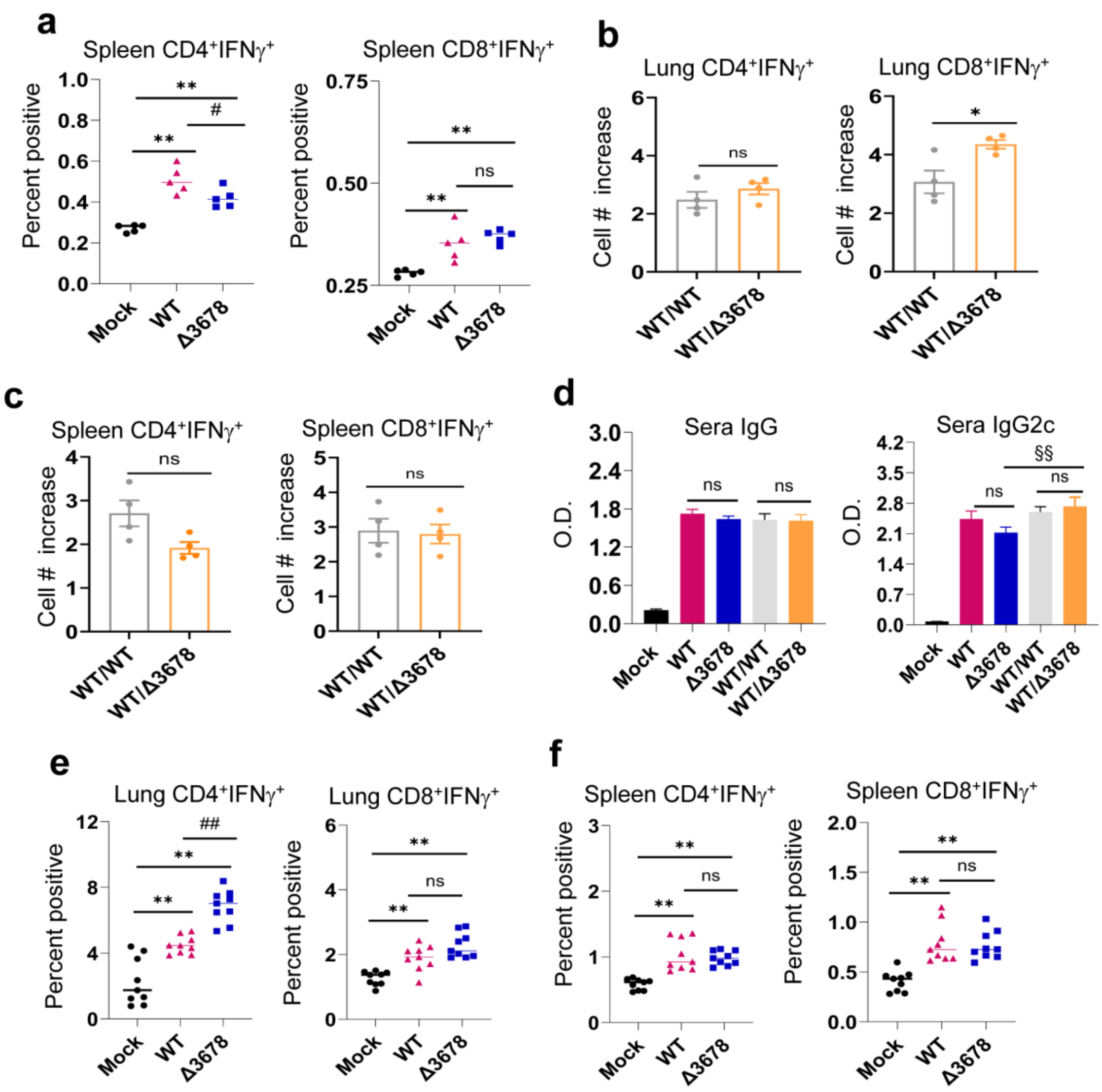

Supplementary Figure 1. SARS-CoV-2 Δ3678 mutant induced strong mucosal and systemic T cell responses in K18-hACE2 mice with or without prior SARS-CoV-2 infection. At 1 month post vaccination, splenocytes of mice without prior SARS-CoV-2 infection (**a**), lung leukocytes (**b**) or splenocytes (**c**) of mice with prior SARS-CoV-2 infection were cultured *ex vivo* with S peptide pools for 5 h, and stained for IFN-γ, CD3, and CD4 or CD8. Percent positive (a) or fold increase of IFN-γ^+ $CD4^+$ and $CD8^+$ T cells expansion compared to the mock vaccinated group (b-c) among lung or spleen T cells is shown. **d.** Sera IgG and IgG2c titers in vaccinated mice with or without prior SARS-CoV-2 infection measured by ELISA. **e-f.** Lung leukocytes (**e**) or splenocytes (**f**) of vaccinated mice

without prior SARS-CoV-2 infection were harvested at day 4 post challenge with WT virus and cultured *ex vivo* with S peptide pools for 5 h, and stained for IFN-γ, CD3, and CD4 or CD8. Percent positive of IFN-γ^+ $CD4^+$ and $CD8^+$ T cells among lung or spleen T cells is shown. ** $P < 0.01$, or *$P < 0.05$ compared to mock group. $^{\#\#}P < 0.01$, or $^{\#}P < 0.05$ compared to WT group. $^{\S\S}P < 0.01$ compared to mice without prior SARS-CoV-2 infection. Unpaired, 2-tailed Student's t test was used to determine the differences. Data are presented as means ± standard error of the mean (s.e.m).

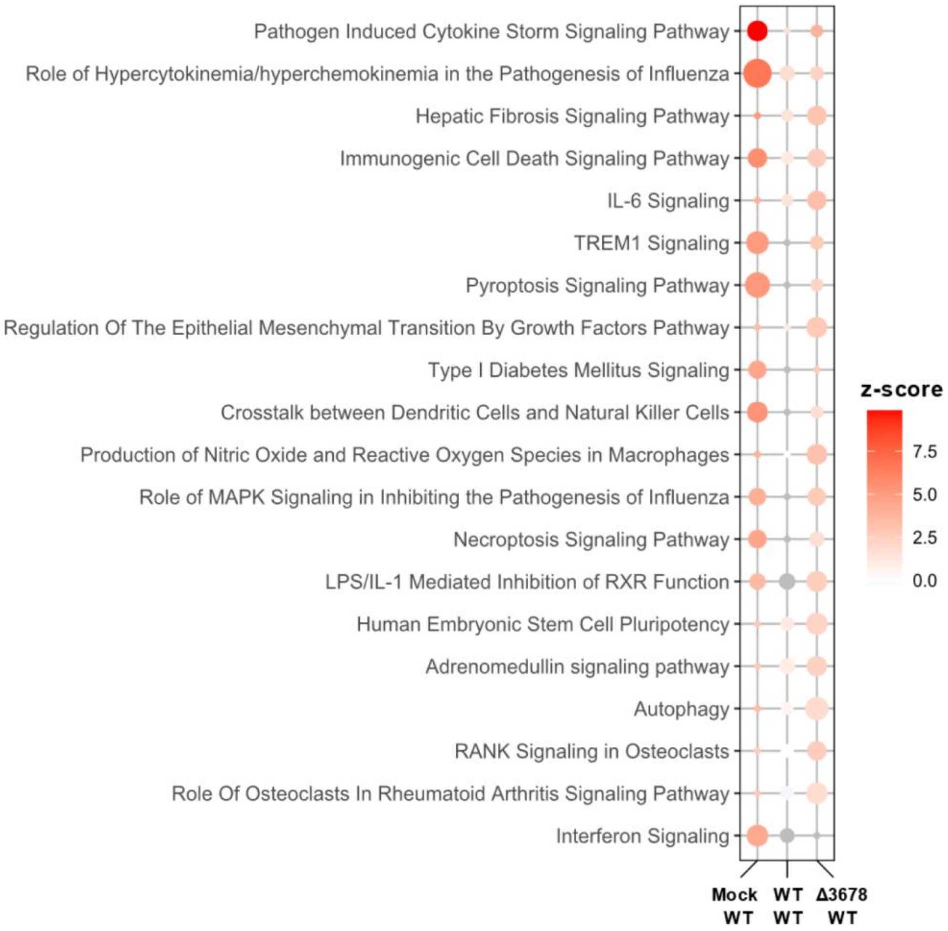

Supplementary Figure 2. NanoString data for mouse whole lung day 4 post-challenge in the vaccinated mice. All comparisons are between a challenged group and unvaccinated/unchallenged (mock/mock) controls. Bubble plot of 20 canonical pathways ordered by ascending p value from comparison analysis in Ingenuity Pathway Analysis. Dot size corresponds to –log(p value) where larger dots indicate more significant p values. Color intensity corresponds to activation z-score and all pathways are either activated or demonstrate no change. Gray indicates values that could not be calculated.

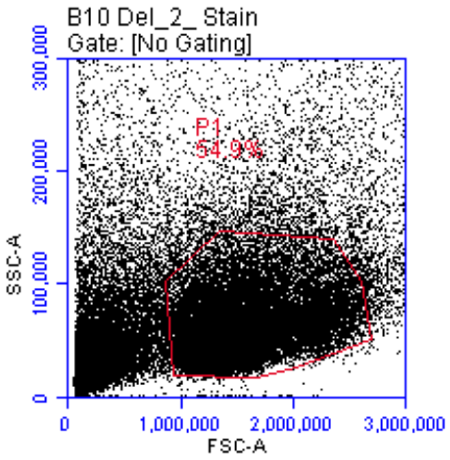

Supplementary Figure 3. Gating Strategy: Samples were acquired with a C6 Flow Cytometer instrument. Total splenocytes were gated. Dead cells were excluded on the basis of forward and side light scatter.

SUPPLEMENTARY METHOD:

Vaccination studies: 8 to10-week-old female heterozygous K18-hACE2 C57BL/6J mice were infected intranasally (i.n.) with 2 x 10^3 PFU of WT WA1 or mock-infected with DPBS followed by vaccination with the same dose of WT WA1 or Δ3678 mutant. At 32 DPV, terminal serum, lungs, and spleens were harvested for analysis.
